# Integrating Creative Approaches to Dementia Care Into Nursing Programmes: Findings From an International Qualitative Study

**DOI:** 10.1111/scs.70131

**Published:** 2025-10-03

**Authors:** Cristina Tommasini, Stefanos Mantzoukas, Charlotte Buitenkamp, Nina van der Vaart, Iris Zampira, Elpiniki Laiou, Athina Paschou, Eftychia Lazarou, Marina Makri, Minna Kuvajainen, Virpi Liljeström, Neel de Haan, Maaike van Ooijen, Magda Tsolaki, Stefania Chiappinotto, Alvisa Palese, Lucia Cadorin

**Affiliations:** ^1^ Department of Medicine Udine University Udine Italy; ^2^ Department of Nursing University of Ioannina Ioannina Greece; ^3^ Department of Innovation National Foundation for the Elderly Amersfoort the Netherlands; ^4^ Greek Association of Alzheimer's Disease and Related Disorders Thessaloniki Greece; ^5^ Department of Health Care and Social Services LAB University of Applied Sciences Lahti and Lappeenranta Finland; ^6^ Department of Institute of Social Work Hogeschool Utrecht the Netherlands; ^7^ 1st Neurology Department of Aristotle, AHEPA Hospital University of Thessaloniki Thessaloniki Greece

**Keywords:** creative approaches, nursing education thematic analysis, older people, qualitative design

## Abstract

**Introduction:**

The literature suggests that the use of creative methods (e.g., art, theatre, and music) in the care of older people can promote interactions and positive experiences. However, there are no studies that have explored the perspectives of nursing programmes internationally regarding their inclusion in the curriculum. Therefore, to expand knowledge from a multinational perspective, this study explored (a) the views and perceptions and (b) the potential barriers to the inclusion of creative approaches in nursing education in the development of competences for the care of people with dementia.

**Methods:**

A qualitative descriptive study according to the Consolidated criteria for Reporting Qualitative research checklist. Semi‐structured face‐to‐face and focus group interviews with a purposive sample of 79 participants in four countries (Finland, Greece, Italy, and the Netherlands). Participants were nursing students (*n* = 32), educators/professors (*n* = 17), healthcare professionals (*n* = 13), family members (*n* = 8), policy makers (*n* = 6), and people with dementia (*n* = 3). The interviews were transcribed verbatim and analyzed using thematic analysis.

**Results:**

Views, perceptions, and barriers/advantages related to creative approaches were summarised into four themes: (a) Being already familiar with creative approaches; (b) Perceiving their potentialities; (c) Bolstering the personalised approach in nursing education; and (d) Ensuring preparedness and support.

**Conclusions:**

The inclusion of creative approaches in nursing education appears to be both mature and desired by stakeholders at the international level, given their diverse potentialities. These approaches are also seen as an excellent way to improve the teaching of personalised care. However, resources as well as a particular attitude and the ability of professors to act as role models are required.

## 1 Introduction

The European population is ageing, and more people are living longer. The number of individuals affected by dementia is thus also increasing from 7.7 million in 2001; it is projected to rise to 15.9 million in 2040 [1], thus increasing the demand for caring services [2]. At the same time, there is a growing shortage of healthcare professionals (HCPs) for elder care, due in part to the unattractiveness of and negative attitudes towards gerontological nursing, especially among younger nurses [3].

To promote the interest of future nurses in the care of older individuals, policy, practice, and research worldwide have developed and evaluated various measures to improve the experience of trainees in nursing homes and other gerontological contexts [4, 5]. Nevertheless, nursing students report that they have no interest in this form of care due to negative stereotypes and a feeling of insecurity [6], which suggests that the gap between supply and demand in this field will increase [7, 8]. Counteracting the negative attitude towards older individuals is thus crucial [9]; part of this negativity is related to the perception of being unprepared to work with older people due to their complex health problems, which require advanced skills not sufficiently developed in undergraduate education [10]. Intergenerational contact programmes that include structured activities to engage different generations [11] may stimulate students' interest in this field and equip them with effective skills [12]. In addition, studies have shown that the use of creative approaches (e.g., art, drama, and music) in older people's care can develop constructive interactions and positive practice experiences that can help to change students' attitudes and work preferences [7], promote enthusiasm, learning, and motivation [13] towards older people's care [10]. Recently, innovative academic educational strategies have been recommended to emphasize the relevance of care in these areas. Faculty have also been recommended to implement innovative methods to instill in students a desire to become involved in the care of the older population [14].

Although nursing programmes usually include an overload of factual scientific information limiting space in the curriculum for more creative types of knowledge, higher education institutions (HEIs) across Europe are becoming aware of and interested in implementing creative approaches and connecting future professionals to older individuals with dementia [3]. Available examples have relied on the Böhm care method, which promotes a broader and more in‐depth understanding of innovative care by exploring the experiences and biography of the older person to highlight family activities with significant emotional repercussions [15]. The subsidised *Long Live Arts* programme in the Netherlands reported on the influence of creative approaches in triggering memory among older individuals with dementia, notably through the Veder contact method, which uses theatrical elements, poetic and musical communication to motivate contact with older people in care settings.

Alongside the potential impact on students' attitudes towards older people, creative approaches can promote person‐centred relationships. Creative approaches encompass different strategies, such as the arts of movement, dance, photography, song, and music. All these complementary interventions can help develop healing relationships and a creative bond for further expression and connection [16]. Music can facilitate significant interactions between caregivers and their loved ones living with dementia, thus relieving tension and providing greater emotional support; the relationship between older individuals with dementia and HCPs may also be positively affected [17]. Instruction on how to involve patients with dementia in creative activities can support interpersonal communication and influence the quality of care through the better identification of needs [18]. Overall, the inclusion of creative arts in caring relations may provide future nurses the capacity to change from *doing* to *just being* with older people with dementia [18, 19]. Creative elements can also motivate students while reinforcing the set of skills required in this field [13]. Evidence has linked creative activities such as writing poems, composing songs, and drawing to the development of students' critical thinking, creativity, and empathic qualities [20]. These strategies can also motivate students to develop fun and meaningful learning, analyze and synthesize fundamental knowledge, and understand the needs of patients and families [13].

Despite their acknowledged relevance, a few studies have explored the perspectives of students, educators, policy makers, and people with dementia and their families in relation to the introduction of such creative approaches in the nursing programs [21]. In addition, no data have been documented on the opportunities and barriers to incorporating these methods into the formal curriculum [22]. Also, no studies have been conducted at a multinational level. Therefore, to expand knowledge from a multinational perspective, this study explored (a) the views, perceptions, and (b) the benefits and barriers to incorporating creative approaches into nursing education aimed at developing competences for the care of people with dementia. Ultimately, the aim was to answer the following research question: “What are the perceptions, views, potential barriers and benefits of incorporating a creative approach into the nursing curriculum from the perspective of *key stakeholders* at a multinational level?” Understanding their perspective can inform educational reforms at local, national, and European levels on how creative approaches can be taught and learned in nursing programmes. By creative approaches, we have here understood all strategies that engage people with dementia in a range of creative arts‐based interventions (e.g., dance, drama, music, art, and poetry) to promote their full participation and the person's remaining strengths, ultimately affirming personhood in the nursing approach [23].

## 2 Methods

### 2.1 Study Design

A descriptive qualitative design [24] according to the Consolidated criteria for Reporting Qualitative (COREQ) research checklist [25] (Table S1) was performed as part of a large European (EU) project named Intergenerational CONtact between studeNts and people with dEmentia through CreaTive education (iConnect). This project was intended at promoting the social inclusion of individuals with dementia and to develop creative approaches to motivate students to choose this setting of care as a career option. To achieve this main intent, a consortium including six organisations from four EU countries was established (the Netherlands, the National Foundation for the Elderly, the Hogeschool Utrecht; Finland, the LAB University of Applied Sciences; Greece, the University of Ioannina, the Alzheimer Hellas; and Italy, the University of Udine).

### 2.2 Participants

A purposive sampling method [26] was used. The consortium members agreed that the ‘key stakeholders in curriculum design’ were people from within and outside the nursing programme whose perspectives, needs, and experiences could provide valuable insight into the field [27]. Therefore, individuals meeting the following criteria were eligible: (1) HEI students aged > 18 years and (2) HEI educators/professors (hereafter, professors) teaching health sciences disciplines; (3) HCPs working with older people with cognitive impairments/dementia, either in a nursing home or home‐care setting; (4) policymakers influencing HEIs' curriculum development policies; (5) family and/or formal or informal caregivers of older individuals in an advanced state of dementia; and (6) older individuals with dementia, either diagnosed or still in the process of diagnosis. Each member of the consortium was responsible for identifying and involving potential participants by contacting accessible HEIs, Alzheimer's organisations, and healthcare facilities. It was agreed to involve at least eight students in each country, whereas a pragmatic approach regarding other participants was used, thus leaving each partner to approach the number of key informants considered relevant and accessible; moreover, sufficient time to involve participants and to achieve thematic saturation [28] at the partner level was ensured. The gradual involvement of stakeholders was decided at the national level, where a representative member was appointed as the person responsible for contacting potential participants and providing the required information about the study process. All those contacted agreed to participate, and no refusals were documented. The process was terminated when saturation was reached at the national level [29]—as agreed by at least two members of the research project. In addition, patients with dementia were only included in one country: This was agreed in the study process according to their accessibility, the competences of the team, and previous research experience in this field.

### 2.3 Data Collection

A semi‐structured interview guide was developed using the literature available [30, 31] during a workshop held in a meeting involving all partners. The questions were then translated into the national languages and piloted in each country.

The interview was organised according to the participant groups: the focus group method was deemed more appropriate for the nursing students, while the other participants were interviewed face‐to‐face to gain access to a wide range of views and increase the richness of the data [32]. The main areas to investigate related to the research aim were considered in all interviews (Table 1).

**TABLE 1:** Stakeholder group: Data collection methods and areas under investigation.

Stakeholder group	Interview method	Country^a^				Specific areas under investigation			
		Finland	Greece	Italy	Netherlands
Familiarity with creative approaches	View/perceptions/experiences creative approaches	Potentialities/effectiveness	Barriers/issues
Students	Focus group	8	8	8	8	√	√	√	√
Professors	Face‐to‐face	4	4	4	5	√	√	√	√
HCPs	Face‐to‐face	0	4	2	7	√	√	√	√
HEI policy makers	Face‐to‐face	2	1	1	2	√	√	√	√
Relatives	Face‐to‐face	0	4	0	4	√	√	√	√
Person with dementia	Face‐to‐face	0	3	0	0	√	√	√	√

Abbreviations: HCPs, Health Care Professionals; HEI, Higher Educational Institution.

^a^ Interviews and focus groups number.

All interviews were conducted in a room agreed upon by the participants to ensure privacy and quiet. The responses and the entire interviews were recorded and transcribed verbatim in the participants' national languages. All transcriptions were double‐checked for accuracy and saturation [29]. They were then translated into English following appropriate criteria [33] to enable analysis and discussion.

The interviewers were primarily female professors in the partner universities and associations, with a higher level of education (ranging from Master to Doctoral degrees) in different disciplines. They were also all experts in conducting interviews. Participants did not know the researchers before the data collection, and no repeated interviews were performed.

### 2.4 Data Analysis and Rigour

A thematic analysis was carried out [34]. The first level of analysis was conducted at country level using an inductive approach. Each partner group, involving at least two members, selected the main significant quotations and sorted a preliminary set of codes; these were sent to two researchers (NV, NDH) who read and re‐read the transcribed quotations and codes several times to familiarise themselves with the content and ensure reliability. This began the second level of analysis: to obtain a holistic description of the phenomenon, the data collected in each stakeholder group (students, professors, policy makers, HCPs, family members, and people with dementia) were triangulated, and the quotes and preliminary codes were clustered into subthemes and then into themes based on similarities and differences [35]. At the third level of analysis, the identified themes were then shared and discussed in a research meeting until consensus was reached on their overall consistency. Sub‐themes were then also identified and agreed upon by all members. The raw analysis was then sent to the partners to assess the saturation of each theme and subtopic at country level [29]; the process was finalised with a consensus meeting.

The analysis pathway was shared and reviewed in advance to ensure consistency of data. Most of the information from the interviews was retained (raw material, codes, and concepts—data available from the authors) to ensure credibility [36].

### 2.5 Ethical Consideration

The ethics committees and/or relevant institutions in each participating partner approved the research protocol. As part of the study, all participants were informed verbally and in writing about the aims and procedures of the study. All participants voluntarily signed the informed consent form, as did the legal representative for persons suffering from dementia. All participants also stated that they agreed that the interview would be recorded. Confidentiality was ensured and participants were free to terminate their participation at any time without consequences.

## 3 Results

### 3.1 Participants

A total of 79 participants (Table 2) were involved; most were students (*n* = 32), followed by professors (*n* = 17), HCPs (*n* = 13), relatives (*n* = 8), policymakers (*n* = 6), and people with dementia (*n* = 3). Students were enrolled primarily in nursing and social work departments, while professors were involved in HEIs as nursing educators, researchers, or supervisors in different fields, including geriatrics. The HCPs were working in an Alzheimer's organisation (*n* = 4), home care and daycare services (*n* = 4), a well‐being organisation (*n* = 3), or a nursing home (*n* = 2). Policymakers were education managers or faculty heads. Individuals with dementia were married, cared for by a spouse, and previously employed in different fields. Two relatives were caring for a partner living with dementia, while six were a parent or grandparent of individuals living with dementia: among them, five were in late‐stage Alzheimer's, one in early stage, and one was recently deceased.

**TABLE 2:** Participants' qualification at the time of the interview (*n* = 79).

Participants (*n*)	Gender^a^	Age^b^	Education^a^			
	Female		Bachelor	Master	PhD	Post higher education
Students^c^ (32)	27 (84.3)	26 (20–51)	—	—	—	—
Professors (17)	14 (82.3)	43 (35–60)	1 (6.0)	10 (58.5)	4 (23.5)	2 (12)
Health care professionals (13)^d^	12 (92.3)	49 (20–67)	10 (77)	2 (15.5)	1 (7.5)	—
Relatives (8)	6 (75)	53 (26–73)	Not requested	Not requested	Not requested	Not requested
HEI policy makers (6)	3 (50)	54 (44–64)	—	—	5 (84)	1 (16)
People with dementia (3)	2 (67)	79 (74–84)	Not requested	Not requested	Not requested	Not requested

Abbreviation: HEI, Higher Educational Institution.

^a^ Number (%).

^b^ Average (range).

^c^ Study years: 1st year (6); 2nd year (17); 3rd–last year (9); Program: Nursing (17); Social work (10); Dram/music therapy (4); Physiotherapy (1).

^d^ Health Care Professionals Qualification: coordinator/manager of a care center (*n* = 6); Case manager or advisor within a care organisation devoted to older individuals (*n* = 3); Psychologist (*n* = 2); Occupational therapist (*n* = 1); home care team (*n* = 1).

Most students reported that they had personal experience with dementia, primarily within their family; they were unsure whether they wanted to work with people with dementia. However, some reported that it would be beneficial for their professional career because of the increasing population in nursing homes who have symptoms of dementia.

Four professors worked directly with people with dementia. Five had personal experience with dementia in their family, while the rest had no direct experience, but their teaching was focused on the field. Most focused on learning to connect directly or indirectly with these people using creative approaches. Relatives indicated that they could still connect with their loved one affected by dementia through touch, walking, or engaging in creative activities, such as looking at family photos. Two relatives reported that contact was no longer possible; one had a difficult relationship with his/her relative with dementia because of refusal behaviours.

At the time of the interviews, the use of these approaches was still relatively new in Greece and Italy, while in Finland and the Netherlands, such approaches had already been incorporated as a standard teaching practice.

### 3.2 The Themes

The views, perceptions, and barriers/benefits associated with creative approaches were summarised into four themes: (a) already familiar with creative approaches; (b) perceptions of their potential; (c) strengthening the personalised approach to nursing education; and (d) ensuring preparedness and support (Table 3).

**TABLE 3:** Themes, subthemes and quotations from the different stakeholder groups.

Themes	Sub‐themes	St	Pr	HCP	HEIPM	Re	PwD	Quotations
Being already familiar with creative approaches	They are part of my life	*●*	*●*				*●*	“Drama, poetry, singing, dancing, musical instruments, poetry, storytelling, games, photography, nature.” arts.” (St 3, 4, 6, 7, 11, 12, 16, 17) “I engage in these activities as hobbies, apart from role playing and groups.” (Pr 11, 12) I like all of these activities. Mostly I like dancing. I follow a program with physical exercises. I really like theatre and cinema, listening to music and dancing.” (PwD 1, 2, 3)
	They are part of my learning and professional experience	*●*		*●*				“Routine, do things they like (listen to music, watch films, go for a walk, painting), cooking, baking, get the family involved, get in touch with friends, bring them things they liked (e.g., food), theatre. Physical contact, smell, music. Everything to stimulate the senses.” (St 2, 17) “We implement music therapy, book therapy, art therapy and many more. There is a theatre group and at the end of each year we arrange a performance. Furthermore, we organise events for the public to raise awareness in Alzheimer's disease and introduce the organisation's work. For example, every year we exhibit mosaics or sculptures that our patients have prepared.” (HCP 4)
Being aware of their multiple potentialities	Creating a connection with students first		*●*		*●*			“Be inspirited and connected to these people. Sensitivity, Attachment, steady personality, Knowledge of artistic forms. As a teacher you must be an exemplary role. Knowledge of dementia and arts, skills of arts. Language of artistic forms.” (Pr 2, 3) “Arts Therapies in all kinds of courses.” (HEIPM 3)
	Creating a connection with patient later	*●*	*●*	*●*				“Gaming: we use games a lot, mostly games for memory training but also to activate people who cannot speak well.” (St 1) “My aunt. She likes to sing and if we sang some songs, she was happy again. Singing gives you a focus. And a way to remember, reminiscing.” (Pr 3) “Very positive to be able to reach people with this, words do not do the trick anymore. Because words fade away, but this reaches to the memories that are still there. Not verbally. For people with first stage dementia, it could be something to get into contact with each other, instead of trying to get the student to get in touch with them. It is not suitable for them. But as a student that helps them to perform themselves and to get in touch with them.” (HCP 10)
	Considering effects also beyond the patient		*●*			*●*		“Singing is a great way to contact people with dementia. It was very helpful for my mother.” (Pr 1) “For me it is important that my father experience something that he likes and shows that he likes it. My father likes music, and he reacts cheerfully to it. The nurses in his home also try to attract cheerful reactions with music of plays on television.” (Re 5)
Bolstering the personalised approach	Continuously assessing the preferences		*●*			*●*	*●*	“My mother… she passed away… I also gave some advice about how to use creative methods with her, for the caregivers in the home, like developing a Life‐book. I also gave them the toolkit for elderly people, the one I created myself with HASIC” and “My aunt. She likes to sing and if we sang some songs, she was happy again. Singing gives you a focus. And a way to remember, reminiscing.” (Pr 3) “The relationship is good. It has improved since she was diagnosed and now, I know how to properly manage her behaviour, most of the time” (Re 1) “No, I wouldn't. I don't think I would be able to participate. I like watching others but I cannot do the same because I don't have the talent.” (PwD 3)
	Preparing to early identify and managing the adverse effects			*●*		*●*		“The students need to be taught how to remain calm and react to unexpected and aggressive behaviour of the patient. They must be prepared and not panic.” (HCP 1) “It depends on the person's mood on each specific day, because it is unpredictable.” (Re 3)
	Adapting the strategies over the time	*●*				*●*		“My two grandmothers. The one still alive has a very small voice nowadays. She speaks very softly now and became very unsociable, which was a big change….” (St 1) “We have a good relationship but her condition is worsening every day so we cannot have a normal conversation.” (Re 3)
Ensuring preparedness and support	Reflecting on some prerequisites			*●*		*●*		“Treat people like you wish to be treated by others. Be sensitive and empathetic” (HCP 7) “Be empathetic, see what you get. No patronising. Feel comfortable to touch someone or react to the reaction” (Re 7)
	Being trained on methods		*●*					“It is necessary to be educated as a creative trainer. When you don't have didactic skills, it is also possible, but can be very difficult.” (Pr 1)
	Being courageous		*●*					“Open‐mindedness, ability to be present (physically and mentally), ability to throw oneself into using this method, courage.” (Pr 9)
	Being motivated and connected with my/your self		*●*					“It depends on the motivation and connection one has towards these activities. One should be attached and feel comfortable in these elements. It should be a natural way to work with this because you can't force being creative. Besides one should believe in these methods to teach and convince others.” (Pr 3)
	Being exemplary and coherent		*●*					“Be inspirited and connected to these people. Sensitivity, Attachment, steady personality, Knowledge of artistic forms. As a teacher you have to be an exemplary role. Knowledge of dementia and arts, skills of arts. Language of artistic forms.” (Pr 2, 3)
	Being supported by appropriate resources		*●*					“We need resources, working hours, colleagues.” (Pr 7)

Abbreviations: HCP, Health Care Professional; HEI PM, Higher Educational Institution Policy Makers; Pr, Educator/Professor; PwD, people with dementia; Re, relative; St, student.

### 3.3 Already Familiar With Creative Approaches

Participants were already familiar with a creative approach as they ‘Are part of my life*’* and ‘Are part of my learning or professional experience’. At the time of the interviews, many of the participants enjoyed numerous activities during their free time—ranging from singing and music to arts, drama, poetry, dancing, sports, drawing, or memory games—as activities that formed a part of their life. The professors were also engaged in music, singing, dancing, sports, poetry, visual arts, drama, photography, and storytelling in their personal time. Most activities were largely hobbies, but some also used them in their teaching. From the point of view of people with dementia, dancing was a general interest, and they all listened to music and enjoyed theatre.

Students and HCPs reported that these activities were also part of their learning and professional experience. Students participated in a wide variety of learning modules, ranging from music to arts and drama activities. The HEI curriculum entailed drama, arts, sports, music, dancing, storytelling about clinical cases, movies, and essays. Photography and advanced simulation were also used. In the Netherlands, creative approaches were used as an inherent part of the curriculum, while in Italy and Greece, they were used as support for communications and interactive courses and in Finland as an element of social work and physiotherapy. Respondents believed that any student could take a course that includes creative elements, although the activities need to match their specific interests. Some HCPs also indicated that they were already using creative approaches in different activities, such as singing, dance, theatre, or intergenerational activities.

### 3.4 Being Aware of Multiple Potentialities

Participants reported the perceived effectiveness of creative approaches in ‘Creating connections with students first,’ ‘Creating connections with patients later,’ and ‘Creating positive effects beyond the patients as well.’ First, students were challenged in understanding their needs of patients with dementia, as well as in facing the behavioural issues. Professors and policymakers thus underlined that using creative approaches in teaching may help students find and use ways to connect with people with dementia. Professors reported that they teach students about ways to connect with people with dementia, both directly and indirectly, using different forms of art, such as drawing or painting.

Second, being equipped with these approaches may help students to create connections with patients later during clinical rotations and in professional life. According to students, it is easier to be in contact with people with dementia by touch, scent, remembering their past, reading books, listening to music, going for a walk, cooking, or looking at family photos. Professors working in the clinical settings underlined that these approaches reflect investments in the capacity of the future workforce to care for these patients. HCPs mentioned that the combined use of creative approaches with more technical or traditional interventions enriches the capacity to take care of these patients in advanced stages of dementia as well, when little interaction may be possible.

Third, the effects are perceived beyond the patient. Relatives of people with dementia wanted their loved ones to experience these emotions in a respectful and pleasant environment because they allow them to feel joy, relax, and have fun. As the disease progresses, these approaches and their effectiveness can also prevent and reduce the negative emotions that relatives experience in the face of their progressive inability to be in contact with their loved ones.

### 3.5 Bolstering the Personalised Approach in Nursing Education

Participants underlined the challenges encountered in providing education regarding personalized care. Teaching creative approaches may bolster the intentions of the nursing programme to expand further their efforts to help students learn how to personalize care effectively by ‘Assessing the patient's preferences, continuously’, ‘Identify early and manage adverse effects’, and ‘Adapt strategies over time’ according to the progression of the disease.

Professors, relatives, and patients reported that not all creative approaches are appropriate and that the individual's preferences and willingness to engage as the disease progresses should be assessed, as needs and preferences may also change. These approaches are not always effective, and according to HCPs and relatives, early recognition of adverse events such as agitation or negative reactions should be carried out by competent nurses. Students should learn how to recognise and deal with unpredictable reactions: patients may not be enthusiastic about interacting with others through theatre—they can feel embarrassed. Some actions (e.g., being asked to sing) can cause agitation, or student participation may be difficult as people with dementia tend to refuse to take part in group activities. HCPs also highlighted that unpredictable and aggressive behaviour of people with dementia can be a significant barrier and that students' lack of understanding of the reactive responses to the proposed method can be a challenge to its application. Given that the progression of the disease may change the individual's preferences and responses, the relatives—as well as the students—emphasised that the creative approaches should be continuously personalised. Teaching the creative approaches in the context of dementia care can therefore further develop the skills to personalise care to the needs of the individual.

### 3.6 Ensuring Preparedness and Support

Participants reported the importance of being prepared and supported in introducing these approaches in nursing education. Specifically, ‘Reflecting on some prerequisites’, ‘Being trained on methods’, ‘*Being courageous*’, ‘Being motivated and connected with my/yourself’, ‘Being exemplary and coherent’, and ‘Being supported by appropriate resources’ were underlined.

Creative approaches cannot be used by everyone. HCPs and patient relatives pointed out that sensitivity, empathy, and affinity are prerequisites for students, professors, and HCPs considering these approaches. Empathy was highlighted as the most important skill students need to connect with older people with dementia. In addition, students and family members pointed out that respect is a necessary skill and that patronizing or banality (e.g., singing) should be avoided.

Creative approaches require education and training. Professors should be trained to incorporate these methods into their daily teaching by acquiring or developing scientific and pedagogical knowledge and skills, accompanied by concrete practical experience (e.g., in theatre). The need to prepare students through appropriate training and to equip them with the underlying thinking and knowledge to feel comfortable and professional in using creative elements in daily care was also emphasised.

Formal education and training are not enough. Professors pointed out that it is important to be bold and open‐minded to incorporate these approaches into formal teaching; it is also important to be motivated and connected to oneself, demonstrating role modelling for students and coherent teaching. Teaching creative approaches requires additional resources—such as time, space, and materials—which should be made available by universities. For example, the group of students participating in creative modules should usually be small (e.g., 15–20). Professors also need supportive colleagues as these approaches complement the care of people with dementia.

## 4 Discussion

To our knowledge, this is the first international qualitative study involving multiple stakeholders to explore the views, perceptions, barriers, and opportunities in relation to creative approaches in nursing education. Overall, four themes emerged: familiarity with creative approaches in personal and professional contexts; recognition of their potential to improve connections and care outcomes; their role in supporting personalised nursing education; and the need for preparation, training, and institutional support to implement them effectively.

First, the findings reflect the views of participants who reported all direct (family members, professional experiences) and/or indirect experiences (e.g., dementia as a field of study and teaching), highlighting their expertise and qualifications on the topic. The presence of creative approaches among hobbies in personal and professional life as well as in the learning pathway was reported by students, professors, HCPs, and people with dementia. This suggests that the topic is already accepted and has overcome some problems at the institutional level that have recently been documented [19]. Millennial nursing students have also been described as creative, multitasking, and collaborative team players who enjoy using active and experiential learning methods in conjunction with technology [37], which may increase positive attitudes towards these approaches.

Secondly, the perceived potential outcomes in three different directions—namely students, patients, and beyond (i.e., relatives and potentially the same HCPs)—suggest that the outcomes are visible and recognized at multiple levels. Potential outcomes were reported in a cascade fashion, suggesting that HEIs implementing these creative approaches may see the initial benefits in students through to carers, depending on how well they are able to apply these approaches in everyday practice. At the student level, it was emphasized that teaching art raises awareness of their potential, gives them more enjoyment in their future work, and enables better care of older people [38]. At the patient level, creative approaches have been used to treat behavioral and psychological symptoms of people with dementia [16, 39], to improve social interaction and integration, communication and contact, to evoke memories and enhance well‐being and quality of life [16] and to strengthen the connection with these patients [40]. Positive effects have also been demonstrated for the relatives of dementia patients [41]. Although the personalized approach is a fundamental principle in nursing education, the use of creative approaches can enhance this skill in students as the disease progression of dementia changes the patient's needs and responses over time. Their inclusion in training therefore appears to reinforce the basic principles of personalized care. Overall, the various possibilities that have emerged here suggest that multiple levels of outcomes should be considered when evaluating the effectiveness of such approaches, even if specific outcomes should not be the focus (e.g., prevention of anxiety, prevention of cognitive decline; [42]). In addition, these approaches may also actively promote the so‐called relational model of citizenship emphasizing the importance of relationships and social interaction in developing engaged and responsible citizens [43].

Fourth, the recommended formal training should be based on personal prerequisites that fall within the domain of moral competences, such as empathy and humanistic sensitivity [43, 44]. These seem to form a fundamental basis from which creative approaches can be fostered with appropriate training in content and methods. The recommended formal training suggests that these approaches cannot be improvised either in universities or in practice: They should be considered like all other nursing interventions and supported by rationale, evidence, appropriate skills labs, and clinical education to avoid trivialisation or inappropriate delivery. A core curriculum is proposed that outlines the requirements of such training.

Finally, instructors teaching creative approaches should be excellent [45]. They should be prepared for the rational basis of creative approaches and have time to prepare for it. They should also be able to flexibly adapt their teaching methods to encourage the development of creative skills [22]. They should create a safe environment for students and encourage their self‐confidence and motivation [37]. They should also act as role models for their students (e.g., being kind when teaching about kindness; making contact when teaching about outreach opportunities). Overall, these salient features may be particularly necessary because of the barriers that still exist in some higher education environments [46] or because of the need to negotiate appropriate resources [21] and ensure the availability of contextual factors that are important while implementing new solutions [16, 47].

Overall, most of the themes were supported by professors, students, and HCPs. Only one theme came from policy makers, and a few from relatives and people with dementia. Whilst the latter is to be expected given the challenges of including people with dementia in research [48], this lack of input from policy makers should be considered a potential area for improvement given their role in shaping the educational environment, vision, and development.

### 4.1 Limitations

This study has several limitations. Firstly, a purposive sample was used, with the profile of participants varying from country to country. For example, in one country (Greece), older people with dementia and in another (Greece and the Netherlands), family members were included. Furthermore, to avoid additional burden, the clinical condition of the person with dementia was not analysed, suggesting that future studies could include a more balanced group of people with dementia, while more data on their profile is proposed. Second, the narratives were translated into English, and although this process was conducted according to guidelines in the field [33], it may have altered the meaning intended by the participants. Third, different methods were used to collect data, although the interviews and the focus groups with students were combined in the data analysis, as described in the literature [32]. However, this process may have influenced the results.

## 5 Conclusion

The inclusion of creative approaches in the education of nursing students appears to be a mature concept and is desired by many stakeholders. Not only are these approaches an integral part of people's lives—especially the new generations of students—but their familiarity can also prevent potential barriers. Students and patients, but also relatives themselves, as well as HCPs and educators, recognize the opportunities that such approaches offer.

The need to apply these approaches, taking into account the evolution of the needs of patients with dementia and preferences, is also seen as an excellent opportunity to improve the teaching of personalised care, which is an essential element and value of the nursing profession. In terms of concrete application in educational practise, time, environmental conditions, and structural resources—but also a particular attitude and the ability of the professor to lead by example—are needed to fully integrate such approaches into the curricula in a coherent way. Therefore, policy makers concerned with educational reform should consider these approaches, while educational institutions should document their implementation to also measure their impact on changes in newly graduated nurses in terms of their career intentions and person‐centred attitudes, which are the long‐term desired outcomes.

## Author Contributions

Study's inception and design: C.T., M.O., N.H., N.V., S.M.; Data acquisition (conducted the interviews/focus group): A. Paschou, C.T., I.Z., E. Lazarou, E. Laiou, M.K., M.M., N.H., N.V., V.L.; Data analysis: A. Paschou, E. Lazarou, E. Laiou, I.Z., L.C., M.K., M.O., N.H., V.L., A. Palese, S.C.; Manuscript development: A. Palese, C.T., L.C., S.C.; Critical revision and supervision of the manuscript: A. Palese, A. Paschou, C.B., E. Laiou, I.Z., M.T., S.M. All authors read and approved the final manuscript.

## Ethics Statement

The 37th Meeting of the Scientific and Ethical Committee of the Greek Association of Alzheimer's Disease and Related Disorders approved the project (21 February 2018). All partners also received approval from their own Ethical Committees or Internal Review Boards.

## Consent

The study provided all participants with oral and written information about the study aims and processes, and all participants voluntarily signed the informed consent form and the legal representative for those individuals with dementia. All participants also declared that they knew the interview would be audio‐recorded; confidentiality was ensured, and participants were left free to terminate their participation at any point without consequence.

## Conflicts of Interest

The authors declare no conflicts of interest.

## Supporting information


**Table S1:** COnsolidated criteria for REporting Qualitative research checklist (COREQ) [25].

## Data Availability

The data that support the findings of this study are available from the corresponding author upon reasonable request.

## References

[1] I. Bacigalupo , F. Mayer , E. Lacorte , et al., “A Systematic Review and Meta‐Analysis on the Prevalence of Dementia in Europe: Estimates From the Highest‐Quality Studies Adopting the DSM IV Diagnostic Criteria,” Journal of Alzheimer's Disease 66 (2018): 1471–1481. 10.3233/JAD-180416 PMC6294583 30412486

[2] A. Cura Della Redazione , “Quando la solitudine e l'isolamento sociale diventano un fattore di rischio per la salute [When loneliness and social isolation become a health risk factor],” Assistenza Infermieristica e Ricerca 42, no. 2 (2023): 57–59. 37309656 10.1702/4050.40311

[3] R. Darling , M. Sendir , S. Atav , and F. Buyukyilmaz , “Undergraduate Nursing Students and the Elderly: An Assessment of Attitudes in a Turkish University,” Gerontology & Geriatrics Education 39, no. 3 (2018): 283–294. 28353413 10.1080/02701960.2017.1311883

[4] M. McAllister , C. Ryan , L. Dodd , M. Goldenberg , and D. L. Brien , “A Thematic Literature Review of Innovative Strategies to Prepare Nursing Students for Aged‐Care,” Nurse Education Today 87 (2020): 104355. 32062413 10.1016/j.nedt.2020.104355

[5] K. L. Rush , S. Hickey , S. Epp , and R. Janke , “Nurses' Attitudes Towards Older People Care: An Integrative Review,” Journal of Clinical Nursing 26, no. 23–24 (2017): 4105–4116. 28639384 10.1111/jocn.13939

[6] K. P. Frausing and A. S. Stamp , “Making a Difference: Students' Experiences With a Dementia Care Program,” Gerontology & Geriatrics Education 42, no. 1 (2021): 126–139. 31442104 10.1080/02701960.2019.1659256

[7] A. Zisberg , M. Topaz , and T. Band‐Wintershtein , “Cultural‐ and Educational‐Level Differences in Students Knowledge, Attitudes, and Preferences for Working With Older Adults: An Israeli Perspective,” Journal of Transcultural Nursing 26, no. 2 (2015): 193–201. 24848351 10.1177/1043659614526252

[8] A. C. Goodyear , A. Arola , and S. Rosendahl , “I Wish I Had Asked for Support Earlier' – Immigrant Family Caregivers' Experiences of Living With a Person With Dementia,” Scandinavian Journal of Caring Sciences 37, no. 3 (2023): 710–719. 36808759 10.1111/scs.13155

[9] F. Riva‐Rovedda , A. Conti , and E. Viottini , “Le conseguenze delle restrizioni delle visite nelle strutture sanitarie residenziali: una revisione narrativa della letteratura [The Consequences of Visiting Restrictions in Long Term Care Facilities: A Narrative Review of the Literature],” Assistenza Infermieristica e Ricerca 42, no. 2 (2023): 82–97. 37309659 10.1702/4050.40314

[10] B. J. King , T. J. Roberts , and B. J. Bowers , “Nursing Student Attitudes Toward and Preferences for Working With Older Adults,” Gerontology & Geriatrics Education 34, no. 3 (2013): 272–291. 23383875 10.1080/02701960.2012.718012 PMC3659195

[11] J. Forster , E. Tullo , L. Wakeling , and R. Gilroy , “Involving Older People in Inclusive Educational Research,” Journal of Aging Studies 56 (2021): 100906. 33712091 10.1016/j.jaging.2020.100906

[12] M. Kimzey , B. Mastel‐Smith , and A. Seale , “Effects of Dementia‐Specific Education for Nursing Students,” Nurse Educator 44, no. 6 (2019): 338–341. 30557205 10.1097/NNE.0000000000000623

[13] J. Rankin and V. Brown , “Creative Teaching Method as a Learning Strategy for Student Midwives: A Qualitative Study,” Nurse Education Today 38 (2016): 93–100. 26775032 10.1016/j.nedt.2015.12.009

[14] V. Consolo , S. B. Clark , and M. P. Lippe , “Undergraduate Nursing Students' Perceptions of Geriatric Care and Nursing Curriculum,” Nurse Educator 50, no. 3 (2025): E175–E180. 39672201 10.1097/NNE.0000000000001789

[15] T. Fertalova and I. Ondriova , “Non‐Pharmacological Treatment of Alzheimer's in Redirecting Alzheimer Strategy – Tracing Memory Loss to Self Pathology,” (2019), https://www.intechopen.com/books/redirecting‐alzheimer‐strategy‐tracing‐memory‐loss‐to‐self‐pathology/non‐pharmacological‐treatment‐of‐alzheimer‐s.

[16] W. Q. Koh , S. A. Felding , K. B. Budak , E. Toomey , and D. Casey , “Barriers and Facilitators to the Implementation of Social Robots for Older Adults and People With Dementia: A Scoping Review,” BMC Geriatrics 21, no. 1 (2021): 351. 34107876 10.1186/s12877-021-02277-9 PMC8191065

[17] R. Rio , “A Community‐Based Music Therapy Support Group for People With Alzheimer's Disease and Their Caregivers: A Sustainable Partnership Model,” Frontiers in Medicine 5 (2018): 293. 30460236 10.3389/fmed.2018.00293 PMC6232122

[18] E. Broome , T. Dening , J. Schneider , and D. Brooker , “Care Staff and the Creative Arts: Exploring the Context of Involving Care Personnel in Arts Interventions,” International Psychogeriatrics 29, no. 12 (2017): 1979–1991. 28879836 10.1017/S1041610217001478

[19] G. Windle , K. Algar‐Skaife , M. Caulfield , et al., “Enhancing Communication Between Dementia Care Staff and Their Residents: An Arts‐Inspired Intervention,” Aging & Mental Health 24, no. 8 (2020): 1306–1315. 30884963 10.1080/13607863.2019.1590310 PMC7446032

[20] X. Ma , Y. Yang , X. Wang , and Y. Zang , “An Integrative Review: Developing and Measuring Creativity in Nursing,” Nurse Education Today 62 (2018): 1–8. 29274494 10.1016/j.nedt.2017.12.011

[21] C. A. Surr , S. Parveen , S. J. Smith , et al., “The Barriers and Facilitators to Implementing Dementia Education and Training in Health and Social Care Services: A Mixed‐Methods Study,” BMC Health Services Research 20, no. 1 (2020): 512. 32503536 10.1186/s12913-020-05382-4 PMC7275489

[22] H. Y. Liu , I. T. Wang , N. H. Chen , and C. Y. Chao , “Effect of Creativity Training on Teaching for Creativity for Nursing Faculty in Taiwan: A Quasi‐Experimental Study,” Nurse Education Today 85 (2020): 104231. 31765871 10.1016/j.nedt.2019.104231

[23] S. Bellass , A. Balmer , V. May , et al., “Broadening the Debate on Creativity and Dementia: A Critical Approach,” Dementia (London) 18, no. 7–8 (2019): 2799–2820. 29631495 10.1177/1471301218760906

[24] M. Sandelowski , “Whatever Happened to Qualitative Description?,” Research in Nursing & Health 23, no. 4 (2000): 334–340. 10940958 10.1002/1098-240x(200008)23:4<334::aid-nur9>3.0.co;2-g

[25] A. Tong , P. Sainsbury , and J. Craig , “Consolidated Criteria for Reporting Qualitative Research (COREQ): A 32‐Item Checklist for Interviews and Focus Groups,” International Journal for Quality in Health Care 19, no. 6 (2007): 349–357. 17872937 10.1093/intqhc/mzm042

[26] M. Q. Patton , Qualitative Research and Evaluation Methods (SAGE Publications, 2015).

[27] E. Belita , N. Carter , and D. Bryant‐Lukosius , “Stakeholder Engagement in Nursing Curriculum Development and Renewal Initiatives: A Review of the Literature,” Quality Advancement in Nursing Education 6, no. 1 (2020): 2.

[28] M. Hennink and B. N. Kaiser , “Sample Sizes for Saturation in Qualitative Research: A Systematic Review of Empirical Tests,” Social Science & Medicine 292 (2022): 114523. 34785096 10.1016/j.socscimed.2021.114523

[29] B. Saunders , J. Sim , T. Kingstone , et al., “Saturation in Qualitative Research: Exploring Its Conceptualization and Operationalization,” Quality and Quantity 52, no. 4 (2018): 1893–1907. 29937585 10.1007/s11135-017-0574-8 PMC5993836

[30] B. Dicicco‐Bloom and B. F. Crabtree , “The Qualitative Research Interview,” Medical Education 40, no. 4 (2006): 314–321. 16573666 10.1111/j.1365-2929.2006.02418.x

[31] K. Peters and E. Halcomb , “Interviews in Qualitative Research,” Nurse Researcher 22, no. 4 (2015): 6–7. 10.7748/nr.22.4.6.s2 25783145

[32] S. D. Lambert and C. G. Loiselle , “Combining Individual Interviews and Focus Groups to Enhance Data Richness,” Journal of Advanced Nursing 62, no. 2 (2008): 228–237. 18394035 10.1111/j.1365-2648.2007.04559.x

[33] N. A. Yunus , T. olde Hartman , P. Lucassen , et al., “Reporting of the Translation Process in Qualitative Health Research: A Neglected Importance,” International Journal of Qualitative Methods 21 (2022): 16094069221145282.

[34] M. Vaismoradi , H. Turunen , and T. Bondas , “Content Analysis and Thematic Analysis: Implications for Conducting a Qualitative Descriptive Study,” Nursing & Health Sciences 15, no. 3 (2013): 398–405. 23480423 10.1111/nhs.12048

[35] D. H. Grossoehme , “Overview of Qualitative Research,” Journal of Health Care Chaplaincy 20, no. 3 (2014): 109–122. 24926897 10.1080/08854726.2014.925660 PMC4609437

[36] I. Korstjens and A. Moser , “Series: Practical Guidance to Qualitative Research. Part 4: Trustworthiness and Publishing,” European Journal of General Practice 24, no. 1 (2018): 120–124. 29202616 10.1080/13814788.2017.1375092 PMC8816392

[37] J. D. Chaudhuri , “Stimulating Intrinsic Motivation in Millennial Students: A New Generation, a New Approach,” Anatomical Sciences Education 13, no. 2 (2020): 250–271. 31021529 10.1002/ase.1884

[38] G. A. Dingle , L. S. Sharman , Z. Bauer , et al., “How Do Music Activities Affect Health and Well‐Being? A Scoping Review of Studies Examining Psychosocial Mechanisms,” Frontiers in Psychology 12 (2021): 713818. 34566791 10.3389/fpsyg.2021.713818 PMC8455907

[39] C. J. Domínguez‐Chávez , B. C. Salazar‐González , and C. J. Murrock , “Use of Music Therapy to Improve Cognition in Older Adults With Dementia: An Integrative Review,” Research and Theory for Nursing Practice 33, no. 2 (2019): 183–195. 31123162 10.1891/1541-6577.33.2.183

[40] A. Creech , “Using Music Technology Creatively to Enrich Later‐Life: A Literature Review,” Frontiers in Psychology 10 (2019): 117. 30761052 10.3389/fpsyg.2019.00117 PMC6363696

[41] C. Bailey , R. Jones , S. Tiplady , I. Quinn , J. Wilcockson , and A. Clarke , “Creative Writing and Dementia Care: ‘Making It Real’,” International Journal of Older People Nursing 11, no. 4 (2016): 244–254. 26786862 10.1111/opn.12113

[42] G. M. Masika , D. S. F. Yu , and P. W. C. Li , “Visual Art Therapy as a Treatment Option for Cognitive Decline Among Older Adults. A Systematic Review and Meta‐Analysis,” Journal of Advanced Nursing 76, no. 8 (2020): 1892–1910. 32201968 10.1111/jan.14362

[43] R. Faggioli , “A Value‐Creating Approach. Dialogues With the Advisory Board: Interview With Namrata Sharma,” In GLOCITED – Editorial Series on Global Citizenship Education (2023), https://unescochairgced.it/en/glocited/a‐value‐creating‐approach/.

[44] J. Wiisak , M. Stolt , M. Igoumenidis , et al., “Factors Contributing to the Promotion of Moral Competence in Nursing,” Nursing Ethics 31, no. 8 (2024): 1367–1388. 38504620 10.1177/09697330241235305 PMC11577688

[45] J. R. Bacsu , Z. Rahemi , D. Petrovsky , et al., “Advancing Gerontology Through Exceptional Scholarship (AGES): A Mentorship Initiative for Early Career Faculty,” Canadian Geriatrics Journal 27, no. 1 (2024): 80–84. 38433883 10.5770/cgj.27.700 PMC10896206

[46] K. D. Velzke , “Exploration of Choice for Older People With Daily Care Needs: Scottish Professionals' Perspectives on Self‐Directed Support,” Journal of Gerontological Social Work 60, no. 1 (2017): 7–31. 27937968 10.1080/01634372.2016.1267674

[47] T. D. Steinskog , O. Tranvåg , D. Ciliska , and B. Graverholt , “Contextual Influences on Knowledge Translation Capacity in a Nursing Home Organisation: A Phenomenological Hermeneutical Study,” Scandinavian Journal of Caring Sciences 38, no. 3 (2024): 767–781. 38666453 10.1111/scs.13264

[48] M. Roes , M. M. Heppner , S. Teupen , et al., “Involving People With a Frontotemporal Dementia Diagnosis in Dementia Research,” Innovation in Aging 6, no. Suppl 1 (2022): 37.

