# Supplementary material for: Integrating Creative Approaches to Dementia Care Into Nursing Programmes: Findings From an International Qualitative Study
Source: Scand J Caring Sci. 2025 Oct 3;39(4):e70131. doi: 10.1111/scs.70131 (PMC12492466; doi:10.1111/scs.70131)
Supplement: Supplementary file 1 — Table S1: COnsolidated criteria for REporting Qualitative research checklist (COREQ) [25]. [file SCS-39-0-s001.docx]

**Supplementary Table 1.** COnsolidated criteria for REporting Qualitative research checklist (COREQ) [25].

| **Topic, Item No.** | | **Guide Questions/Description** | | **Brief description** | |
| --- | --- | --- | --- | --- | --- |
| **Domain 1: Research team and reﬂexivity** | | | | | |
| *Personal characteristics* | | | | | |
| Interviewer/facilitator | 1 | | Which author/s conducted the interview or focus group? | | Methods  *Data collection* |
| Credentials | 2 | | What were the researcher’s credentials? e.g. PhD, MD | | Methods  *Data collection* |
| Occupation | 3 | | What was their occupation at the time of the study? | | Methods  *Data collection* |
| Gender | 4 | | Was the researcher male or female? | | Methods  *Data collection* |
| Experience and training | 5 | | What experience or training did the researcher have? | | Methods  *Data collection* |
| *Relationship with participants* | | | | | |
| Relationship established | 6 | | Was a relationship established prior to study commencement? | | Methods  *Participants* |
| Participant knowledge of  the interviewer | 7 | | What did the participants know about the researcher? e.g. personal goals, reasons for doing the research | | Methods  *Participants* |
| Interviewer characteristics | 8 | | What characteristics were reported about the interviewer/facilitator? e.g. Bias, assumptions, reasons and interests in the research topic | | Methods  *Data collection* |
| **Domain 2: Study design** | | | | | |
| *Theoretical framework* | | | | | |
| Methodological orientation and  Theory | 9 | | What methodological orientation was stated to underpin the study? | | Methods  *Design* |
| *Participant selection* | | | | | |
| Sampling | 10 | | How were participants selected? e.g. purposive, convenience,  consecutive, snowball | | Methods  *Participants* |
| Method of approach | 11 | | How were participants approached? e.g. face-to-face, telephone, mail, email | | Methods  *Participants*  *Table 1* |
| Sample size | 12 | | How many participants were in the study? | | Results |
| Non-participation | 13 | | How many people refused to participate or dropped out? Reasons? | | Methods  *Participants*  Results |
| Setting | | | | | |
| Setting of data collection | 14 | | Where was the data collected? e.g. home, clinic, workplace | | Methods  *Data collection* |
| Presence of non-  participants | 15 | | Was anyone else present besides the participants and researchers? | | Methods  *Data collection* |
| Description of sample | 16 | | What are the important characteristics of the sample? e.g. demographic data, date | | Results  *Table 2* |
| *Data collection* | | | | | |
| Interview guide | 17 | | Were questions, prompts, guides provided by the authors? Was it pilot tested? | | Methods  *Data collection* |
| Repeat interviews | 18 | | Were repeat inter views carried out? If yes, how many? | | Methods  *Data collection* |
| Audio/visual recording | 19 | | Did the research use audio or visual recording to collect the data? | | Methods  *Data collection* |
| Field notes | 20 | | Were field notes made during and/or after the interview or focus group? | | Methods  *Data collection* |
| Duration | 21 | | What was the duration of the interviews or focus group? | | NA |
| Data saturation | 22 | | Was data saturation discussed? | | Methods  *Data analysis* |
| Transcripts returned | 23 | | Were transcripts returned to participants for comment and/or correction? | | Methods  *Data analysis* |
| **Domain 3: Analysis and ﬁndings** | | | | | |
| *Data analysis* | | | | | |
| Number of data coders | 24 | | How many data coders coded the data? | | Methods  *Data analysis* |
| Description of the coding  tree | 25 | | Did authors provide a description of the coding tree? | | NA |
| Derivation of themes | 26 | | Were themes identified in advance or derived from the data? | | Methods  *Data analysis* |
| Software | 27 | | What software, if applicable, was used to manage the data? | | Methods  *Data analysis* |
| Participant checking | 28 | | Did participants provide feedback on the findings? | | Methods  *Data analysis* |
| *Reporting* |  | |  | |  |
| Quotations presented | 29 | | Were participant quotations presented to illustrate the themes/findings? Was each quotation identified? e.g. participant number | | Results |
| Data and findings consistent | 30 | | Was there consistency between the data presented and the findings? | | Results |
| Clarity of major themes | 31 | | Were major themes clearly presented in the findings? | | Results  *Table 3* |
| Clarity of minor themes | 32 | | Is there a description of diverse cases or discussion of minor themes? | | Results  *Table 3* |
